# Supplementary material for: A functional screen of RNA binding proteins identifies genes that promote or limit the accumulation of CD138+ plasma cells
Source: eLife. 2022 Apr 22;11:e72313. doi: 10.7554/eLife.72313 (PMC9106329; doi:10.7554/eLife.72313)
Supplement: Transparent reporting form [file elife-72313-transrepform1.pdf]

Westbrook Centre
Milton Road
Cambridge CB4 1YG, UK

P 01223 855340
W elifesciences.org
T @elife

eLife's transparent reporting form

We encourage authors to provide detailed information *within their submission* to facilitate the interpretation and replication of experiments. Authors can upload supporting documentation to indicate the use of appropriate reporting guidelines for health-related research (see EQUATOR Network), life science research (see the BioSharing Information Resource), or the ARRIVE guidelines for reporting work involving animal research. Where applicable, authors should refer to any relevant reporting standards documents in this form.

If you have any questions, please consult our Journal Policies and/or contact us: editorial@elifesciences.org.

Sample-size estimation

- You should state whether an appropriate sample size was computed when the study was being designed
- You should state the statistical method of sample size computation and any required assumptions
- If no explicit power analysis was used, you should describe how you decided what sample (replicate) size (number) to use

Please outline where this information can be found within the submission (e.g., sections or figure legends), or explain why this information doesn't apply to your submission:

No specific determination of sample size was performed before starting this experiment. Instead, we relied on previous experimental results which indicated the numbers of animals used would detect large differences. Some chimeras did not reconstitute properly and therefore were not immunised. For our µMT chimeric mouse experiment our sample size was 6 control mice and 8 knockout mice. For our competitive chimeric mouse experiment our sample size was 3 control mice and 6 knockout mice; moreover, each mouse was reconstituted with CD45.1 YTHDF2 sufficient cells so our competitive chimeric mouse experiment had in total 9 such CD45.1 internal controls. Our experimental design involved 6 mice in each group at every readout, however, some animals failed to respond at all to NP-KLH, which met our exclusion criteria, thus our actual sample sizes were smaller as indicated in the figure legends. Our RNA-seq experiment had a sample size of three mice. This information is given in the figure legends, and in the materials and methods section.

Replicates

- You should report how often each experiment was performed
- You should include a definition of biological versus technical replication
- The data obtained should be provided and sufficient information should be provided to indicate the number of independent biological and/or technical replicates
- If you encountered any outliers, you should describe how these were handled
- Criteria for exclusion/inclusion of data should be clearly stated
- High-throughput sequence data should be uploaded before submission, with a private link for reviewers provided (these are available from both GEO and ArrayExpress)

Please outline where this information can be found within the submission (e.g., sections or figure legends), or explain why this information doesn't apply to your submission:

Our high-throughput sequencing data is all available through GEO, this is described in our manuscript subsection: Materials and data availability. Our CRISPR/Cas9 genetic screen was performed twice on separate occasions with cells from one mouse (biological and technical replicates). Our m6A-eCLIP was performed once with sample prepared from two different mice in parallel (biological replicates). Our RNA-seq experiments was performed once with cells prepared from three different mice (biological replicates). Our sgRNA validation experiments for proliferation/survival (Rc3h1) was performed once. Our sgRNA validation experiments for CD138+ cell accumulation were performed between 2 and 4 times. Each validation experiment involved multiple unique sgRNAs targeting each target gene in Cas9-expressing B cells prepared from a single mouse. Each repeat of the experiment involved the same sgRNA sequences, freshly generated retrovirus, and a unique donor Cas9 mouse (biological replicates). Our competitive chimera experiment was performed once, our µMT readout was performed twice , each mouse was a biological replicate.

eLife Sciences Publications, Ltd is a limited liability non-profit non-stock corporation incorporated in the State of Delaware, USA, with company number 5030732, and is registered in the UK with company number FC030576 and branch number BR015634 at the address Westbrook Centre, Milton Road, Cambridge CB4 1YG.

Statistical reporting

- Statistical analysis methods should be described and justified
- Raw data should be presented in figures whenever informative to do so (typically when N per group is less than 10)
- For each experiment, you should identify the statistical tests used, exact values of N, definitions of center, methods of multiple test correction, and dispersion and precision measures (e.g., mean, median, SD, SEM, confidence intervals; and, for the major substantive results, a measure of effect size (e.g., Pearson's r, Cohen's d)
- Report exact p-values wherever possible alongside the summary statistics and 95% confidence intervals. These should be reported for all key questions and not only when the p-value is less than 0.05.

Please outline where this information can be found within the submission (e.g., sections or figure legends), or explain why this information doesn't apply to your submission: (For large datasets, or papers with a very large number of statistical tests, you may upload a single table file with tests, Ns, etc., with reference to sections in the manuscript.)

The statistical tests used are described in figure legends. For mouse experiments we used two-tailed unpaired Student's t-tests to compare whether the means of two groups were statistically significantly different from a null hypothesis. These groups were normally distributed and independent of each other. Analysis of our genetic screens was performed with the MAGeCK software. The non-targeting negative control sgRNAs were used to build a mean-variance model for null distribution, which in turn was used to calculate significant sgRNA enrichment. Positive and negative enrichments were calculated, then independently used in robust rank aggregation to obtain gene-level scores. This process identified the extent to which each sgRNA enriched out of the distribution of negative control sgRNAs, then ranked genes by the consistency of their sgRNAs to outperform the null hypothesis (that they would not enrich outside the NT-sgRNAs). Gene-level p-values were calculated by randomizing sgRNA to target gene allocation in permutation tests; false discovery was controlled by the Benjamini–Hochberg procedure. Log2 fold changes (LFC) were calculated as the median LFC for all sgRNAs for a targeted gene that enriched in the same direction. Z-scores were calculated as the number of standard deviations from the mean of all negative control LFCs. For m6A data, significant clusters of crosslink sites were identified using CLIPper (https://github.com/YeoLab/clipper/wiki/CLIPper-Home) and significant individual crosslink sites identified using PureCLIP (https://doi.org/10.1186/s13059-017-1364-2), each according to their documented statistical analyses. Z-scores for enrichment of 5mer motifs were calculated as the difference between occurrence at the crosslink sites compared with the mean across 100 sets of randomly generated sites, normalised by the standard deviation across the randomly generated sites. RNA-seq analysis to identify differentially expressed genes was performed with DESeq2 (https://doi.org/10.1186/s13059-014-0550-8) using the documented statistical analysis, with p values adjusted for false discovery rate using the Benjamini-Hochberg method.

Group allocation

- Indicate how samples were allocated into experimental groups (in the case of clinical studies, please specify allocation to treatment method); if randomization was used, please also state if restricted randomization was applied
- Indicate if masking was used during group allocation, data collection and/or data analysis

eLife Sciences Publications, Ltd is a limited liability non-profit non-stock corporation incorporated in the State of Delaware, USA, with company number 5030732, and is registered in the UK with company number FC030576 and branch number BR015634 at the address Westbrook Centre, Milton Road, Cambridge CB4 1YG.

Please outline where this information can be found within the submission (e.g., sections or figure legends), or explain why this information doesn't apply to your submission:
For both the µMT and competitive chimera experiments the mice reconstituted with *Ythdf2*$^{fl/fl}$ and *Ythdf2*$^{fl/fl}$ Vav-iCre bone marrow were housed together in cages of four mice (2 control, 2 KO).

Additional data files ("source data")

- We encourage you to upload relevant additional data files, such as numerical data that are represented as a graph in a figure, or as a summary table
- Where provided, these should be in the most useful format, and they can be uploaded as "Source data" files linked to a main figure or table
- Include model definition files including the full list of parameters used
- Include code used for data analysis (e.g., R, MatLab)
- Avoid stating that data files are "available upon request"

Please indicate the figures or tables for which source data files have been provided:

We have provided source data files for our sgRNA library and our CRISPR/Cas9 genetic screens. For supplementary figure 1 these are supplementary table 1-3 which list the RNA binding proteins targeted by our library, the sequence and representation of sgRNAs in the library, and the enrichment of genes in the proliferation/survival screen. For figure 1 these are supplementary tables 4 and 5 which list the enrichment of genes in the CD138+ cell accumulation screen, and the RBPs which significant enrichment for regulating CD138+ cell accumulation without enrichment for proliferation/survival. For our m6A-eCLIP we attach the coordinates of significant crosslink sites and clusters, as well as the Kmer analysis output for each replicate. For our RNA-seq analysis we attach the DEseq2 output of significant genes.

Figure 4: Z-scores for motifs localising to m6A eCLIP crosslink sites were calculated using the kmer analysis, and sites clusters were annotated for the genes and features to which they map using the iCLIP_Genialis_feature_annotation R scripts, both of which are documented and available on Github (https://github.com/LouiseMatheson/Process_CLIP_data). Visualisation of the m6A distribution across individual genes was performed using a shiny app; the underlying R code and pre-processing script are documented and available on Github (https://github.com/LouiseMatheson/iCLIP_visualisation_shiny).

eLife Sciences Publications, Ltd is a limited liability non-profit non-stock corporation incorporated in the State of Delaware, USA, with company number 5030732, and is registered in the UK with company number FC030576 and branch number BR015634 at the address Westbrook Centre, Milton Road, Cambridge CB4 1YG.
